# Early Detection of Diabetic Cardiomyopathy Using Speckle Tracking Echocardiography: A Systematic Review

**DOI:** 10.7759/cureus.92653

**Published:** 2025-09-18

**Authors:** Mohammad Burhanuddin, Zeeshan Ahmed, Ali Hamza, Muhammad Zeeshan, FNU Abdul Rehman, Syed Hassan M Gillani, Aiman Gul, Zara Rabbani, Osarenoma Mathilda Omonfuegbe, Syed Momin Ali

**Affiliations:** 1 Medicine, Bhaskar Medical College, Hyderabad, IND; 2 Medicine, King Edward Medical University, Lahore, PAK; 3 Medicine, Dow International Medical College, Karachi, PAK; 4 Medicine, Irrua Specialist Teaching Hospital, Irrua, NGA

**Keywords:** cardiac imaging, diabetes mellitus, diabetic cardiomyopathy, early detection, global longitudinal strain, left ventricular function, myocardial strain, speckle tracking echocardiography, subclinical myocardial dysfunction, systematic review

## Abstract

Diabetic cardiomyopathy (DCM) represents a distinct myocardial disease that develops independently of coronary artery disease and hypertension in patients with diabetes mellitus. Early detection of subclinical myocardial dysfunction is crucial for timely intervention and improved patient outcomes, as traditional echocardiographic parameters often remain normal during the initial disease stages. Speckle tracking echocardiography (STE) has emerged as a sensitive, non-invasive imaging technique capable of detecting subtle myocardial deformation abnormalities before conventional parameters become abnormal. This systematic review evaluated the effectiveness of STE in early detection of DCM by analyzing original research studies that assessed myocardial strain parameters in patients with diabetes with preserved left ventricular ejection fraction. A comprehensive literature search was conducted across multiple databases from inception to June 2025, following Preferred Reporting Items for Systematic Reviews and Meta-Analyses (PRISMA) guidelines. Seven studies met the inclusion criteria, encompassing 361 diabetic patients and 300 healthy controls, with publication years ranging from 2013 to 2022. The review included both type 1 and type 2 diabetes mellitus patients with varying disease durations and glycemic control levels. Global longitudinal strain emerged as the most consistently impaired parameter across all studies, demonstrating a significant reduction in patients with diabetes compared to controls despite preserved ejection fraction. Additional strain parameters, including circumferential, radial, and area strains, showed progressive impairment correlating with diabetes duration and glycemic control. Three-dimensional speckle tracking provided enhanced assessment capabilities, revealing more comprehensive myocardial dysfunction patterns. Multi-layered strain analysis demonstrated preferential endocardial involvement, supporting the pathophysiological understanding of subendocardial fiber vulnerability in the hearts of patients with diabetes. Right ventricular strain assessment revealed biventricular involvement in DCM. The findings consistently demonstrated correlations between strain abnormalities and diabetic microvascular complications, suggesting systemic disease involvement. This review provides compelling evidence supporting STE as a valuable tool for early DCM detection, enabling potential early intervention strategies to prevent progression to symptomatic heart failure.

## Introduction and background

Diabetic cardiomyopathy (DCM) is a distinct myocardial disease associated with diabetes mellitus (DM) that occurs independently of coronary artery disease and hypertension [1]. It is characterized by structural and functional changes in the myocardium, leading to impaired cardiac performance. Early DCM often manifests as subclinical myocardial dysfunction, primarily diastolic dysfunction, which can progress to systolic impairment and ultimately heart failure (HF) if undetected and untreated [1]. Given the increasing global prevalence of diabetes and its significant cardiovascular morbidity and mortality, early detection of myocardial involvement in patients with diabetes is critical for timely intervention and improved prognosis.

Traditional echocardiographic parameters such as left ventricular ejection fraction (LVEF) and fractional shortening have limited sensitivity in identifying early myocardial dysfunction in patients with diabetes, as these measurements often remain within normal ranges during the subclinical phase of cardiomyopathy [2]. Consequently, more sensitive imaging modalities are required to detect subtle changes in myocardial mechanics before overt cardiac dysfunction develops. Speckle tracking echocardiography (STE) has emerged as a novel, non-Doppler, angle-independent echocardiographic technique that quantifies myocardial deformation by tracking natural acoustic markers, speckles, within the myocardium throughout the cardiac cycle [3]. By measuring myocardial strain and strain rate in multiple directions (longitudinal, circumferential, and radial), STE provides detailed insights into regional and global myocardial contractility and mechanics. These sensitive parameters can detect subclinical myocardial dysfunction even in patients with preserved LVEF and normal conventional echocardiographic findings [3].

Multiple studies have demonstrated that STE can reveal early myocardial deformation abnormalities in patients with diabetes before the development of clinically apparent cardiomyopathy. For example, global longitudinal strain (GLS), a key STE-derived parameter, is frequently reduced in asymptomatic individuals with diabetes compared to healthy controls, reflecting early impairment of subendocardial fibers that are especially vulnerable to hyperglycemic injury [4-8]. Other deformation indices, including circumferential and radial strains, along with strain rates, also show decrement in the hearts of patients with diabetes, suggesting diffuse myocardial involvement. Importantly, STE-based abnormalities correlate with diabetes duration, glycemic control, and body mass index, indicating their clinical relevance in the assessment of disease progression. Animal model studies using STE provide further evidence for its utility in early diabetic cardiac dysfunction detection. In type 2 diabetic db/db mice, STE-derived radial and circumferential strain abnormalities precede changes in LVEF, capturing early contractile impairment that standard echocardiography cannot detect [9]. Similarly, in type 1 diabetic models, STE parameters identify left ventricular regional dysfunction as early as one week post-diabetes onset, underscoring its sensitivity in revealing early myocardial injury associated with diabetes [10].

The advantages of STE include its reproducibility, angle independence, and ability to assess myocardial mechanics comprehensively in two and three dimensions. Advanced three-dimensional STE (3D-STE) methods may offer improved accuracy in strain measurement and better assessment of complex myocardial deformation patterns, though at the cost of requiring higher image quality and expertise [11]. Early identification of diabetic myocardial dysfunction using STE has important clinical implications. Detecting these subclinical changes may facilitate early lifestyle modifications, aggressive glycemic control, and cardioprotective therapies that could retard or prevent progression to symptomatic HF. Moreover, STE can be instrumental in monitoring therapeutic efficacy over time. However, despite the accumulating evidence supporting the role of STE in early DCM detection, there remains heterogeneity in reported strain values across studies. Factors contributing to this include differences in STE techniques, software algorithms, patient populations, and comorbid conditions. Further standardized, large-scale studies and meta-analyses are required to establish definitive diagnostic thresholds and to better integrate STE into the routine cardiac care protocols for patients with diabetes.

## Review

Materials and methods

Study Selection

This systematic review was conducted in accordance with the Preferred Reporting Items for Systematic Reviews and Meta-Analyses (PRISMA) 2020 guidelines [12]. A comprehensive literature search was performed to identify original research articles, observational studies, and clinical trials assessing the utility of STE for early detection of DCM. The search aimed to capture data on STE parameters as diagnostic or prognostic markers of myocardial dysfunction in patients with diabetes. Five major electronic databases, PubMed, Embase, Scopus, Web of Science, and the Cochrane Library, were systematically searched from database inception up to June 30, 2025. The search strategy combined Medical Subject Headings (MeSH) and free-text keywords related to diabetes mellitus, diabetic cardiomyopathy, and speckle tracking echocardiography. Representative search terms included “diabetic cardiomyopathy,” “diabetes mellitus,” “speckle tracking echocardiography,” “myocardial strain,” “early detection,” and “subclinical cardiac dysfunction.” No language restrictions were applied during the search. Reference lists of all the included articles and relevant reviews were manually screened to identify additional eligible studies not captured by the database search. All search results were imported into Zotero for deduplication, and the deduplicated records were uploaded into the Rayyan platform (Rayyan Systems Inc., Cambridge, MA, US) for systematic screening. Two independent reviewers screened the titles and abstracts to exclude irrelevant records. The full texts of the remaining studies were obtained and assessed against the inclusion criteria. Disagreements were resolved by discussion and, when necessary, adjudicated by a third reviewer.

Eligibility Criteria

Studies were eligible for inclusion if they involved patients with a confirmed diagnosis of Type 1 or Type 2 diabetes mellitus (DM) and explicitly excluded those with overt coronary artery disease or hypertension to isolate the effects of DCM. Included studies were required to evaluate myocardial function using STE, reporting parameters such as GLS, circumferential strain, radial strain, or strain rate. Only studies assessing early or subclinical myocardial dysfunction in patients with diabetes with preserved LVEF or asymptomatic status were considered. Eligible articles comprised original research, including prospective or retrospective cohort studies, cross-sectional studies, and clinical trials, and had to be published in peer-reviewed journals. Exclusion criteria encompassed studies based on animal models or in vitro data, review articles, editorials, grey literature, conference abstracts, or non-systematic narrative reports. Additionally, studies that combined DCM with other cardiomyopathies without providing disaggregated data, or those lacking echocardiographic strain analysis specifically using STE technology, were excluded.

Data Extraction

Data extraction was performed independently by two reviewers using a pre-designed, standardized form capturing essential study characteristics and outcomes. Extracted variables included first author, publication year, study design, sample size, diabetes type and duration, STE parameters assessed (e.g., GLS, circumferential strain), echocardiographic equipment and software used, comparison groups (e.g., diabetic versus healthy controls), and key findings related to myocardial deformation indices. Additional data on correlations with glycemic control, clinical outcomes, and follow-up duration were also recorded. Discrepancies in the extracted data were resolved by consensus or consultation with a third reviewer to ensure accuracy.

Quality Assessment

The methodological quality and risk of bias of included studies were assessed independently by two reviewers using the Newcastle-Ottawa Scale (NOS) [13]. The NOS evaluates domains including selection of study groups, comparability of cohorts, and assessment of outcomes. Each study was rated as high, moderate, or low quality based on these domains. Differences in quality assessment were resolved through discussion.

Data Analysis

Given the heterogeneity in study designs, patient populations, STE parameters, and outcome measures across included studies, a quantitative meta-analysis was not performed. Instead, a narrative synthesis was carried out to summarize the evidence regarding STE’s effectiveness in detecting early myocardial dysfunction in patients with diabetes. Results were qualitatively compared, highlighting consistent patterns in strain abnormalities and their clinical correlates. Potential sources of heterogeneity, methodological limitations, and gaps in the evidence base were critically appraised to inform future research directions.

Results

Study Selection Process

**Figure 1:**
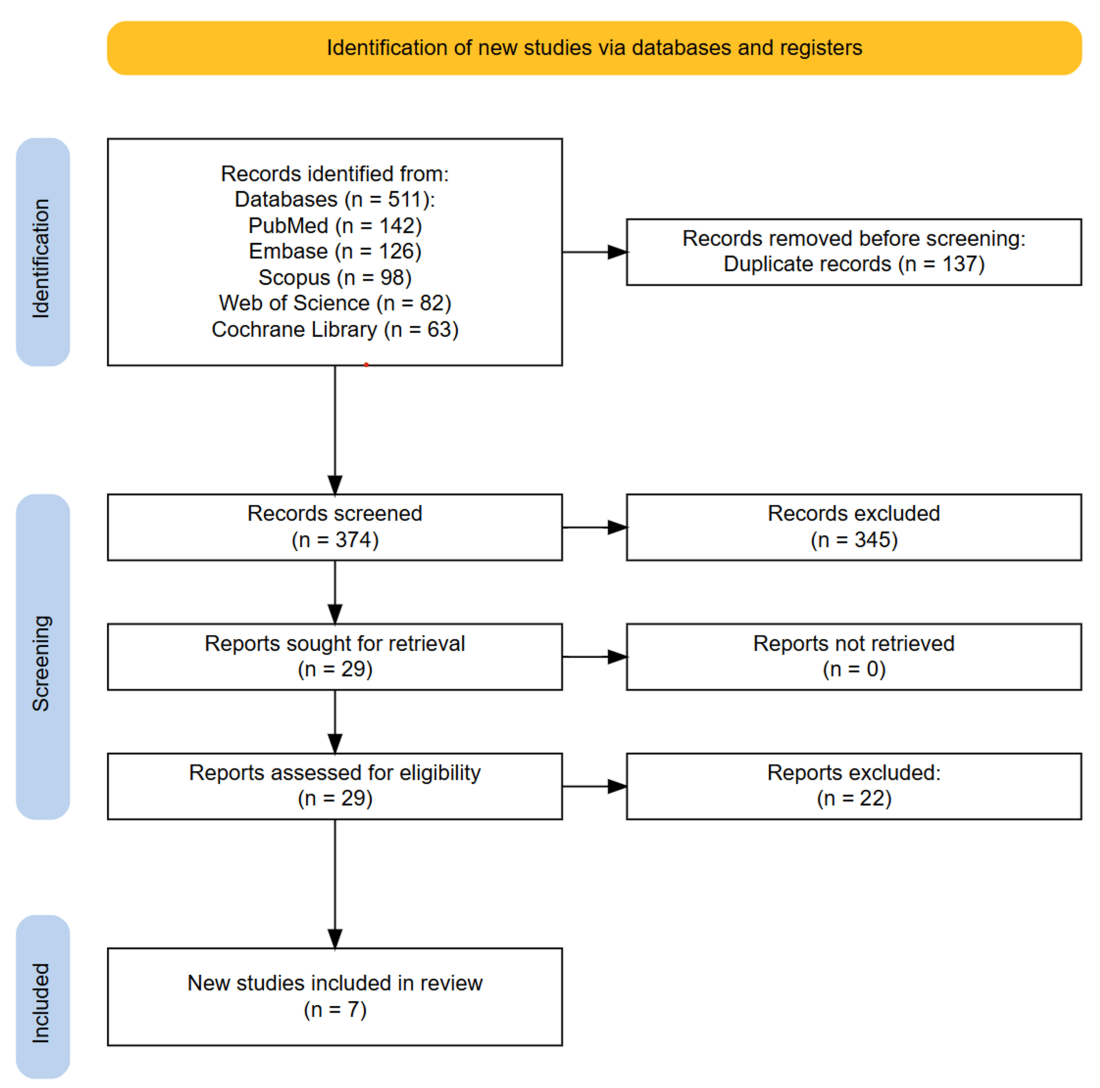
PRISMA diagram illustrating the study selection process PRISMA: Preferred Reporting Items for Systematic Reviews and Meta-Analyses.

A total of 511 records were retrieved through comprehensive searches across PubMed (n=142), Embase (n=126), Scopus (n=98), Web of Science (n=82), and the Cochrane Library (n=63). After the removal of 137 duplicates using Zotero, 374 unique records remained for screening. Two reviewers independently assessed titles and abstracts using the Rayyan web application, resulting in 29 studies selected for full-text review. Of these, 22 articles were excluded for not meeting the predefined eligibility criteria, including studies using non-speckle tracking imaging modalities (n=17), populations not isolated to diabetic cardiomyopathy or mixed cardiomyopathies without subgroup analysis (n=3), and ineligible study design such as case reports, editorials, or reviews (n=2). Ultimately, seven original research articles met all the inclusion criteria and were included in the final analysis. No additional studies were identified through manual reference screening. Any disagreements during the selection process were resolved through discussion without the need for third-party adjudication. The study selection process is summarized in the PRISMA flow diagram (Figure 1).

Study Characteristics

Seven studies met the inclusion criteria for this systematic review, encompassing a total of 361 patients with diabetes and 300 healthy controls. The studies were published between 2013 and 2022, with sample sizes ranging from 30 to 77 participants with diabetes [4-6,8,14-16]. Four studies investigated patients with Type 2 DM, while three focused on Type 1 DM. The mean age of participants varied from 18.2 to 60 years, reflecting different diabetic populations from young adults to middle-aged individuals. The duration of diabetes ranged from newly diagnosed patients to those with long-standing disease (up to median duration of 16 years). Glycemic control varied significantly across studies, with HbA1c levels ranging from well-controlled to poorly controlled. All studies included patients with preserved LVEF, ranging from 56% to 69%, ensuring focus on subclinical dysfunction. Various echocardiographic systems were employed, including the GE Vivid series with EchoPAC software (GE Healthcare, Chicago, Illinois, USA) and the Toshiba Aplio-Artida with Wall Motion Tracking software (Canon Medical Systems, Tochigi, Japan). Both 2D and 3D speckle tracking techniques were utilized, with GLS being the most commonly assessed parameter across all studies (Table 2).

**Table 1:** A summary of the characteristics of the included studies 2DE: two-dimensional echocardiography; 3DE: three-dimensional echocardiography; 3D STE: three-dimensional speckle tracking echocardiography; ACR: area change ratio; CVRR: cardiovascular reflex response; DM: diabetes mellitus; GAS: global area strain; GCS: global circumferential strain; GLEDSR: global longitudinal early diastolic strain rate; GLS: global longitudinal strain; GLSS: global longitudinal systolic strain; GLSSR: global longitudinal systolic strain rate; GRS: global radial strain; HbA1c: glycated hemoglobin; LV: left ventricular; LVEF: left ventricular ejection fraction; RV: right ventricular; RV-GLS: right ventricular global longitudinal strain; STE: speckle tracking echocardiography; T1DM: type 1 diabetes mellitus; T2DM: type 2 diabetes mellitus; TDI: tissue Doppler imaging. [4-6,8,14-16]

Author	Year	Study design	Sample size (diabetes/control)	Diabetes type	Mean age (± SD)	Diabetes Duration (years) Mean or median duration of diabetes	HbA1c (%)	LVEF (%) Mean LVEF	Type of echo used with software/vendor	Outcomes assessed	Main findings	Conclusions
Zhang et al.	2013	Cross-sectional study	68/63 (31 controlled DM + 37 uncontrolled DM/63 controls)	Type 2	Controlled DM: 61±9 years; Uncontrolled DM: 60±10 years	Controlled DM: 7 (4-13) years median; Uncontrolled DM: 8 (5-16) years median	Controlled DM: 6.1±0.5%; Uncontrolled DM: 9.1±1.4%	Controlled DM: 62±5%; Uncontrolled DM: 61±3%; Controls: 63±4.6% (all ≥55%)	3D STE using Vivid E9 (GE Medical Systems) with 4V-D transducer	GLS, GCS, GAS, GRS	Uncontrolled DM patients had significantly reduced peak systolic strain in all directions compared to controls and controlled DM patients. Controlled DM patients showed reduction only in GLS compared to controls. HbA1c was independently associated with GLS, GCS, and GAS.	GLS may be a sensitive indicator of early LV systolic dysfunction in DM patients despite normal LVEF. Poor glycemic control (HbA1c≥7%) leads to reductions in all components of LV systolic strain, independently associated with preclinical LV dysfunction.
Tadic et al.	2014	Cross-sectional study	50/50	Type 2	52±8 years	Not specified (untreated patients)	7.4±0.7%	2DE: 63±4%; 3DE: 62±4%	2D and 3D STE using Vivid 7 (GE Healthcare) with EchoPAC 110.1.2 software	2DE parameters: GLS, GCS, GRS, systolic and diastolic strain rates, LV torsion, and untwisting rate. 3DE parameters: Global longitudinal, circumferential, radial strain, Global area strain	2DE findings: All strain directions were impaired (longitudinal: -20.9±1.7 vs -21.7±1.8%, p=0.025; circumferential: -21.2±2.5 vs -22.4±2.7%, p=0.023; radial: 43.2±11.2 vs 48.3±13.1%, p=0.039). 3DE findings: All 4 strain components significantly decreased (longitudinal: -17.8±2.5 vs -19.1±2.7%, p=0.014), and the LV untwisting rate increased (114±26 vs -96±23°/s, p<0.001)	LV mechanics were impaired in all directions in untreated T2DM patients. HbA1c was independently associated with 3DE LV mechanics. Subclinical changes can serve as early cardiac damage markers. Both 2DE and 3DE can detect early dysfunction before symptomatic disease
Abdel Salam et al.	2016	Cross-sectional study	30/30	Type 1	26.5±4.3 years	14.3±5.8 years	7.4±1.7%	58.2±3.8% (2-D method); 65.5 ± 6.0% (M-mode method)	2D STE using GE Vivid 7 Pro with EchoPac PC software (GE Vingmed Ultrasound, GE Medical Systems, USA)	GLSS, GLSSR, GLEDSR, conventional echocardiographic parameters, tissue Doppler imaging parameters	GLSS was significantly decreased in diabetic patients (-17.7±2.5% vs -21.2±1.7%, p<0.001). GLSSR was decreased (-1.1±0.2 vs -1.3±0.2 s⁻¹, p=0.003). Diastolic dysfunction was evident by conventional echo and TDI. Global longitudinal early diastolic strain rate was comparable between groups (1.5±0.4 vs 1.6±0.3 s⁻¹, p=0.33)	In asymptomatic patients with type 1 DM, GL systolic function measured by speckle-tracking echocardiography was impaired versus controls; diastolic function was impaired by conventional echocardiography and TDI. Early changes in both systolic and diastolic function may be detected in young asymptomatic T1DM with long-standing poorly controlled diabetes
Enomoto et al.	2016	Cross-sectional study	77/35	Type 2	56±15 years	Not explicitly stated	10.6±2.5%	66.3±7.7%	3D STE using Aplio-Artida (Toshiba Medical Systems) with 3D Wall Motion Tracking software	GLS, GCS, GRS, ACR	GLS, GCS, and ACR were significantly impaired in DM patients vs. controls (12.0±3.0% vs. 16.2±1.9%, 27.7±7.1% vs. 32.2±5.7%, and 37.6±7.6% vs. 44.0±6.2%, respectively, p<0.001). GLS was significantly related to CVRR (R=0.58, p<0.001), retinopathy stage, and nephropathy stage. GRS showed no significant difference between groups.	3D-STE-derived longitudinal systolic dysfunction was associated with diabetic microvascular complications, especially cardiac autonomic neuropathy as assessed by CVRR, in asymptomatic non-ischemic patients with poorly controlled T2DM. Diabetic microangiopathy and its accumulated effects were significantly related to subclinical LV dysfunction characterized by impaired longitudinal shortening.
Ahmed et al.	2018	Cross-sectional study	39/15	Type 1	18.2±1.7 years	9.6±3.9 years	8.9±1.7%	69.2±13.1%	2D STE using the Vivid-7GE system with EchoPAC. GE VERSION 110-1-2	RV-GLS, RV systolic and diastolic function, functional capacity	Significantly decreased RV-GLS in the diabetic group (14.0 ± 6.9 vs. 22.7 ± 2.5, p<0.001), decreased RV S velocity (9.5±2.2 vs. 11.5 ± 1.8, p<0.05), reduced E/A ratio (1.0 ± 0.2 vs. 1.1 ± 0.1, p<0.05), and increased E/Em ratio (7.9 ± 3.2 vs. 5.2 ± 0.7, p<0.001). 62% had both impaired RV systolic function and diastolic dysfunction	Young asymptomatic T1DM patients have subclinical RV systolic and diastolic dysfunction despite preserved LVEF and RVEF. 2D-STE can detect subclinical RV systolic dysfunction before conventional echocardiography parameters become abnormal
Berceanu et al.	2019	Cross-sectional study	50/80	Type 1	30.5±4.7 years	9±6 years	8.9±1.6%	60±7% (T1DM group) vs 58±4% (controls)	2D STE using GE Vivid S60 with EchoPAC BT13 workstation	Multi-layered longitudinal strain (endocardial, myocardial, epicardial GLS), RV strain, mechanical dispersion	Significantly reduced endocardial GLS (-20.6±2.7 vs -22.0±2.3, p<0.05) and myocardial GLS (-18.0±2.4 vs -19.1±1.9, p<0.05) in the T1DM group. Higher LV mechanical dispersion (34±11 vs 29±7, p<0.05). No significant RV strain differences.	Young adults with DM1 without known heart disease have subclinical myocardial dysfunction with lower LV endocardial and myocardial longitudinal strain and higher mechanical dispersion demonstrated by multi-layered STE, despite preserved ejection fraction.
Yang et al.	2022	Cross-sectional study	47/27	Type 2	DM A group: 51.42±8.94 years; DM B group: 52.16±9.22 years	DM A group: 9.11±5.19 years; DM B group: 10.36±3.45 years	DM A group: 6.08±0.36%; DM B group: 9.22±0.92%	DM A group: 58.39±2.65%; DM B group: 56.53±7.10%; Control: 59.07±2.32%	2D STE using Vivid E9 GE Medical Systems scanner with EchoPAC workstation (2D-Strain, EchoPAC PC113, GE Healthcare)	GLS, GRS, GCS, torsion parameters (peak twisting, peak twisting velocity, peak untwisting velocity, untwisting rate), conventional echocardiographic parameters	GLS was reduced in both DM groups vs. controls (DM A: -17.25 ± 2.43%, DM B: -15.1±3.22% vs. Control: -20.23±2.45%). GCS reduced in the DM B group (-19.57±3.16% vs. control: -22.1±3.02%). Torsion parameters (peak twisting, untwisting rate) were reduced in DM groups. No significant differences in LVEF between groups	2D-STE can detect subclinical cardiac dysfunction in T2DM patients before conventional parameters show abnormalities. GLS is reduced in early T2DM and decreases further with microvascular complications. Both systolic and diastolic dysfunction can be monitored using strain and torsion analysis

Quality Assessment

The methodological quality of the seven included studies was assessed using the NOS. Overall, the studies demonstrated moderate to high quality, with scores ranging from six to eight out of nine possible points (Table 2).

**Table 2:** Quality assessment of the included studies using the Newcastle-Ottawa Scale [4-6,8,14-16]

Author	Selection (max 4)	Comparability (max 2)	Outcome (max 3)	Total score	Quality rating
Zhang et al.	4	2	2	8	High
Tadic et al.	3	2	3	8	High
Abdel Salam et al.	3	2	2	7	Moderate
Enomoto et al.	4	2	2	8	High
Ahmed et al.	3	1	2	6	Moderate
Berceanu et al.	4	2	2	8	High
Yang et al.	3	2	2	7	Moderate

All the studies scored well in the selection domain, with appropriate case and control definitions and adequate representativeness of study populations. Most studies (six out of seven) achieved maximum scores for comparability, effectively controlling for important confounding factors such as age, hypertension, and coronary artery disease. The outcome assessment domain showed consistent strength across studies, with all employing validated echocardiographic techniques and appropriate statistical methods. The primary limitations identified were related to follow-up duration, as all studies employed cross-sectional designs without longitudinal assessment. Two studies received lower scores due to insufficient description of non-respondents and potential selection bias. Despite these limitations, the overall quality was deemed sufficient for reliable evidence synthesis, with consistent methodology and appropriate statistical analyses supporting the validity of findings.

Discussion

This systematic review demonstrated compelling evidence for the utility of STE in detecting early myocardial dysfunction in patients with diabetes before conventional echocardiographic parameters become abnormal. The consistent finding across all included studies was the significant reduction in GLS in patients with diabetes compared to healthy controls, despite preserved LVEF. This pattern suggests that GLS serves as a sensitive early marker of DCM, capable of identifying subclinical myocardial involvement when traditional parameters remain within normal ranges. The pathophysiological basis for these findings relates to the preferential involvement of subendocardial fibers in DCM. Hyperglycemia-induced metabolic changes, including advanced glycation end products formation, oxidative stress, and microvascular dysfunction, initially affect the subendocardial layer where longitudinal fibers are predominantly located [1]. This explains why longitudinal strain abnormalities precede circumferential and radial strain impairments, as demonstrated in several included studies. The progressive nature of strain abnormalities correlates with diabetes duration and glycemic control, supporting the concept that metabolic control influences the severity of myocardial involvement.

The studies revealed interesting patterns regarding different strain parameters. While GLS was consistently impaired across all studies, circumferential and radial strain showed more variable results. Zhang et al. demonstrated that patients with uncontrolled diabetes exhibited impairment in all strain directions, while those with diabetes under control showed primarily longitudinal strain reduction [14]. This suggests a hierarchical pattern of myocardial involvement, with longitudinal strain being the most sensitive early indicator, followed by circumferential and radial components as the disease progresses. Three studies employed 3D-STE, which provided additional insights into myocardial mechanics [5,6,14]. Tadic et al. and Enomoto et al. demonstrated that 3D-STE could detect more comprehensive strain abnormalities, including area strain and mechanical dispersion parameters [5,6]. The 3D approach offered theoretical advantages by providing more accurate strain measurements through assessment of true myocardial deformation in 3D space, potentially overcoming the limitations of 2D techniques that may be affected by out-of-plane motion.

The clinical implications of subclinical strain abnormalities extend beyond diagnostic utility. Several studies established correlations between strain parameters and diabetic complications, particularly microvascular disease. Enomoto et al. showed significant associations between GLS and cardiac autonomic neuropathy, retinopathy, and nephropathy stages, suggesting that myocardial strain abnormalities may reflect systemic diabetic microangiopathy [5]. This finding supports the concept that cardiac involvement in diabetes is part of a broader microvascular disease process. The review also highlights the potential of STE in monitoring both left and right ventricular function. Ahmed et al. specifically examined right ventricular strain, demonstrating that subclinical right ventricular dysfunction occurs in young patients with Type 1 DM [15]. This biventricular involvement suggests that DCM affects global cardiac function, not just left ventricular mechanics. Multi-layered strain analysis, as demonstrated by Berceanu et al., provides additional mechanistic insights by separately assessing endocardial, myocardial, and epicardial strain components [16]. The finding that endocardial strain is preferentially affected supports the pathophysiological understanding of subendocardial vulnerability in the hearts of patients with diabetes.

The prognostic implications of early strain abnormalities remain an important area for future investigation. While the included studies focused on diagnostic utility, the detection of subclinical dysfunction may facilitate early interventions that could prevent progression to overt heart failure. The ability to monitor therapeutic efficacy using strain parameters could potentially guide treatment optimization in patients with diabetes.

Limitations and future directions

Several limitations were identified in this systematic review that warrant consideration. The heterogeneity in study populations, including different diabetes types, disease duration, and glycemic control levels, limited the ability to establish universal diagnostic thresholds for diabetic cardiomyopathy. Variations in echocardiographic equipment, software versions, and analysis techniques across studies contributed to the differences in reported strain values, highlighting the need for standardization of STE methodology. The cross-sectional design of all the included studies prevented the assessment of longitudinal changes in strain parameters and their prognostic significance. Sample sizes were relatively small, ranging from 30 to 77 patients with diabetes, potentially limiting the generalizability of findings. The exclusion of patients with coronary artery disease and hypertension, while necessary to isolate diabetic cardiomyopathy effects, may not reflect real-world diabetic populations where these comorbidities frequently coexist.

Future research should focus on establishing standardized protocols for STE acquisition and analysis in patients with diabetes, including normal reference ranges for different demographic groups. Large-scale, multicenter prospective studies are needed to determine optimal strain cutoff values for early DCM detection and to assess the prognostic value of strain abnormalities for predicting future cardiovascular events. Longitudinal studies tracking strain changes over time in relation to glycemic control, therapeutic interventions, and clinical outcomes would provide valuable insights into disease progression and treatment efficacy. Investigation of artificial intelligence and machine learning approaches for automated strain analysis could improve reproducibility and clinical implementation. Additionally, research into the cost-effectiveness of routine STE screening in patients with diabetes and its impact on clinical decision-making and patient outcomes is essential for widespread clinical adoption.

## Conclusions

This systematic review provided robust evidence supporting the clinical utility of STE for early detection of DCM in patients with preserved LVEF. GLS consistently emerged as the most sensitive early marker of subclinical myocardial dysfunction, demonstrating significant impairment across all included studies regardless of diabetes type or patient demographics. The progressive nature of strain abnormalities correlated with diabetes duration, glycemic control, and microvascular complications, and underscored the clinical relevance of these findings for disease monitoring and risk stratification. The hierarchical pattern of myocardial involvement, with longitudinal strain abnormalities preceding circumferential and radial impairments, reflected the pathophysiological progression of diabetic cardiomyopathy from subendocardial to transmural involvement. Three-dimensional speckle tracking and multi-layered strain analysis provided enhanced diagnostic capabilities and mechanistic insights into disease progression. Despite demonstrated clinical potential, the standardization of acquisition protocols, establishment of universal diagnostic thresholds, and validation through large-scale prospective studies remain essential for widespread clinical implementation. Future research should focus on determining prognostic value, cost-effectiveness, and optimal integration of STE into routine diabetic care protocols to facilitate early intervention strategies and improve long-term cardiovascular outcomes in patients with diabetes.
